# AUTO-MUTE 2.0: A Portable Framework with Enhanced Capabilities for Predicting Protein Functional Consequences upon Mutation

**DOI:** 10.1155/2014/278385

**Published:** 2014-08-17

**Authors:** Majid Masso, Iosif I. Vaisman

**Affiliations:** Laboratory for Structural Bioinformatics, School of Systems Biology, George Mason University, Manassas, VA 20110, USA

## Abstract

The AUTO-MUTE 2.0 stand-alone software package includes a collection of programs for predicting functional changes to proteins upon single residue substitutions, developed by combining structure-based features with trained statistical learning models. Three of the predictors evaluate changes to protein stability upon mutation, each complementing a distinct experimental approach. Two additional classifiers are available, one for predicting activity changes due to residue replacements and the other for determining the disease potential of mutations associated with nonsynonymous single nucleotide polymorphisms (nsSNPs) in human proteins. These five command-line driven tools, as well as all the supporting programs, complement those that run our AUTO-MUTE web-based server. Nevertheless, all the codes have been rewritten and substantially altered for the new portable software, and they incorporate several new features based on user feedback. Included among these upgrades is the ability to perform three highly requested tasks: to run “big data” batch jobs; to generate predictions using modified protein data bank (PDB) structures, and unpublished personal models prepared using standard PDB file formatting; and to utilize NMR structure files that contain multiple models.

## 1. Introduction

Site-directed mutagenesis experiments provide researchers with opportunities to evaluate their effects on protein stability, activity, or disease potential, to annotate structural or functional roles of residues, to gain insights into mechanisms of protein folding, and to accumulate data needed for engineering new proteins with desired thermodynamic and physicochemical properties. A number of* in silico* mutagenesis methodologies have been developed in recent years [1–5], yielding efficient computational analogues to complement experimental methods from the wet laboratory at a fraction of the time and cost, as well as reliable and immediate predictions for functional effects of single residue replacements [6]. Each approach uniquely employs evolutionary, sequence, or structural information to characterize residue substitutions in proteins, and predictions of functional effects are obtained via mathematical, rule-based, or statistical learning methods.

We previously developed the AUTO-MUTE server, an online set of tools for predicting protein functional consequences upon mutation, by implementing a computational mutagenesis technique that employs a four-body, knowledge-based statistical potential function derived via the coarse graining of protein structures at the amino acid level [7, 8]. For proteins with known 3D structures, any mutation defined by a single residue replacement can be represented as a vector of features that include data derived from our* in silico* mutagenesis procedure. Large sets of diverse mutations that have been studied experimentally for their functional effects, which occur in proteins that share low sequence similarity, were used to train the AUTO-MUTE predictors. Our models were developed by implementing classification and regression statistical machine learning algorithms using the Java-based Weka software package [9].

Here we introduce AUTO-MUTE 2.0, a portable alternative to the web-based server, with platform-specific and command-line driven versions designed for Windows (PC) and Linux/Unix (Mac), as well as for Cygwin, a Unix working environment emulator for Windows (free downloads available from http://proteins.gmu.edu/automute). All of the in-house Java and PHP codes associated with the original online version have been rewritten in the Perl programming language for our new AUTO-MUTE 2.0 stand-alone application, with extensive adjustments introduced into the codes to offer an expanded set of options based upon user feedback about the web server. Necessitating our development of separate versions of AUTO-MUTE 2.0 is its reliance on computational geometry and statistical machine learning software tools freely available for download from outside research groups, respectively known as Qhull (http://www.qhull.org/) [10] and Weka (http://www.cs.waikato.ac.nz/ml/weka/) [9], which are not platform-independent and used by our software without modification.

## 2. Methods

Our approach to predicting protein functional consequences upon single residue replacement focuses specifically on the ensuing local structural effects and begins by identifying, with the use of the Qhull program, all residue positions that are structural neighbors of the residue undergoing mutation. Relevant attributes regarding the mutated position and its six closest neighbors, which include empirical measures quantifying the structural impacts at all of these positions, form the individual component values of a unique feature vector that characterizes the protein mutation. The structural perturbation values are obtained with our* in silico* mutagenesis procedure that relies on a four-body potential energy function we developed, and the feature vectors of the single residue mutations are supplied as input to predictive models that we trained using statistical machine learning algorithms implemented with the Weka software package.

The prediction programs in AUTO-MUTE 2.0 initially convert the user-supplied file of single residue substitution requests, which may occur in any number of distinct protein structures, into a file consisting of their respective feature vectors. This process involves submitting each protein structure to the Qhull program in order to identify the six closest structural neighbors of each residue position undergoing mutation, followed by applying a number of support programs that we prepared (available in a subfolder from the AUTO-MUTE 2.0 download) for determining the attribute values corresponding to each single residue mutation prediction request and generating its feature vector. A file containing all feature vectors for requested mutations is supplied to the AUTO-MUTE 2.0 program selected by the user, and predictions made by the program are presented in an output table. Each AUTO-MUTE 2.0 program utilizes predictive models, trained with large numbers of diverse single residue mutations (similarly represented as feature vectors) whose functional consequences under consideration (stability change, activity change, or human nsSNP disease potential) are experimentally documented, which were developed by implementing machine learning algorithms using Weka. Finally, these trained Weka models (available in another subfolder from the AUTO-MUTE 2.0 download) are called upon to predict the functional consequences of the user-requested single residue mutations.

### 2.1. Four-Body Statistical Potential

**Figure 1:**
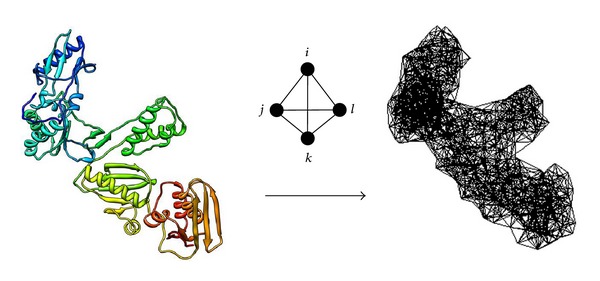
Delaunay tessellation of the HIV-1 reverse transcriptase enzyme (PDB ID: 1rtjA). Initially, the protein is represented as a discrete set of points in 3D space, corresponding to the C-alpha atomic coordinates of every amino acid residue in the structure. A 3D tetrahedral tiling is then obtained by using these C-alpha points to serve as vertices. The complete tessellation yields hundreds of solid tetrahedra that are packed against one another in the form of a convex hull, filling the space otherwise occupied by the protein structure. Shown here is the modified tessellation obtained by removing all edges longer than 12 Å, which reveals clefts and pockets on the protein surface and ensures that each tetrahedron identifies a quadruplet of interacting amino acid residues at its four vertices via their C-alpha coordinates. Each C-alpha point is typically shared as a vertex by several tetrahedra as a result of their packed arrangement; hence, each amino acid may simultaneously participate in a number of distinct nearest neighbor residue quadruplets.

To derive the energy function, we selected X-ray crystallographic structures for 1417 single protein chains (http://proteins.gmu.edu/automute/tessellatable1417.txt) with high resolution (≤2.2 Å), sharing low sequence similarity (<30%), from the protein data bank (PDB) [11]. Each structure is abstracted to a collection of points in three-dimensional (3D) space, corresponding to the C-alpha coordinates of all its constituent amino acid residues (i.e., coarse graining of the protein structure at the residue level). The set of C-alpha points associated with a protein structure are used as vertices to create hundreds of nonoverlapping, space filling, irregular tetrahedra that collectively form a convex hull, referred to as a Delaunay tessellation in the computational geometry literature [12], which we generate with the Qhull software package [10]. Each tetrahedron in the tessellation objectively identifies at its four vertices a quadruplet of nearest neighbor residues in the protein structure; however, as an added measure to exclude false-positive residue quadruplet interactions, all tetrahedral simplex edges longer than 12 Å are immediately removed from every tessellation prior to further analysis [7, 8]. Since the tetrahedra forming a Delaunay tessellation are solid and pack against one another (i.e., two adjacent tetrahedra in a tessellation may share one vertex, one edge—two vertices, or one triangular face—three vertices), each C-alpha point generally serves simultaneously as a vertex for several tetrahedra in the tessellation (Figure 1).

There are 8855 distinct quadruplets of residues that can be generated using the standard 20-letter protein alphabet, by allowing repeated occurrences of residue types in a quadruplet (e.g., CCHH are in close proximity in zinc finger proteins, potentially defining the four vertices of a tetrahedron) while excluding all permutations of any previously listed quadruplets (i.e., the four vertices of each tetrahedron are not ordered). For each type of residue quadruplet (*i*, *j*, *k*, and *l*), let *f*_*ijkl*_ denote the observed proportion of tetrahedra generated by all 1417 protein structure tessellations for which these four residues appear at the vertices, and let *p*_*ijkl*_ represent a rate expected by chance for the same quadruplet, computed using the multinomial probability distribution

(1) $$\begin{array}{c} p_{ijkl}=\frac{4!}{\prod_{n=1}^{20}\left(t_{n}!\right)}\overset{20}{\underset{n=1}{\prod }}a_{n}^{t_{n}}, \end{array}$$

where ∑_*n*=1_^20^ *a*_*n*_ = 1 and ∑_*n*=1_^20^ *t*_*n*_ = 4. Here *a*_*n*_ represents the proportion of all residues comprising the 1417 proteins that are of type *n*, and *t*_*n*_ is the number of occurrences of residue type *n* in the (*i*, *j*, *k*, and *l*) quadruplet. Applying the inverted Boltzmann principle [13], the score *s*_*ijkl*_ = −log⁡(*f*_*ijkl*_/*p*_*ijkl*_) is proportional to the energy of interaction for the quadruplet of residues. The combined set of such scores for all 8855 quadruplet types defines the four-body statistical potential, which is available in the main AUTO-MUTE 2.0 folder under the filename potential_1417_cut12.out. These scores can be used to compute a* total potential* for any protein structure, first by using all its amino acid residue C-alpha points to generate the Delaunay tessellation of the protein (subject to removal of all edges longer than 12 Å) and then by adding up the scores of the residue quadruplets identified at the four vertices of all the constituent tetrahedra in the tessellation. For each amino acid sequence position in a protein, a* residue environment score* is defined by adding up the scores of only those tetrahedra that share the corresponding C-alpha point as a vertex [7, 8].

### 2.2. Computational Mutagenesis

A residue substitution is introduced into a tessellated protein structure by changing the amino acid label at the appropriate C-alpha point. This change alters by a single residue the makeup of quadruplets associated with all tetrahedra that share this point as a vertex. Consequently, the scores of these tetrahedra are changed. Hence, the residue environment score at the mutated position (i.e., the sum of those tetrahedral scores, as defined at the end of the prior section) is altered from wild type, as are the residue environment scores at all structurally nearby sequence positions whose C-alpha points are connected to that of the mutated position by a tetrahedral edge. The nonzero difference (mutant-wild type) between calculated residue environment scores at each of these protein sequence positions is referred to as an* environmental change (EC) score* [7, 8]. Collectively, these nonzero EC scores empirically quantify expected environmental perturbations, due to the residue replacement, at the mutated position itself and at all other (nonmutated) positions within a local 3D neighborhood as defined by the protein tessellation. Hence, our* in silico* mutagenesis approach focuses on carefully modeling mutational impacts at structurally nearby positions, which may be distant from the mutated position in the primary sequence of the protein, while excluding those effects at spatially long-distance positions given the methodological derivation (i.e., each of their EC scores is zero) and yet providing an effective approximation due to the relatively lower influence of the latter with respect to functional prediction.

The residue replacement is subsequently represented as a feature vector, supplied as the input to statistical learning algorithms, that encodes the following information concerning the mutated position: identities of the native and new amino acids, EC score, secondary structure (labeled SS in the prediction output table generated by AUTO-MUTE 2.0; see Table 1 for an example), mean tetrahedrality (labeled sT in the output table), and mean volume (labeled Vol. in the output table) of all tetrahedra that share the mutated position (i.e., its C-alpha point) as a vertex in the tessellation, depth (surface, undersurface, or buried) as defined by the tessellation (labeled Loc. in the output table), and number of tessellation edge connections with surface positions (labeled Num. in the output table). Also encoded in the feature vector is information pertaining to precisely the six residues that are closest to the mutated position in 3D space, as determined by lengths of tessellation edges connecting their respective C-alpha points: amino acid identities, EC scores, and primary sequence locations in the protein relative to that of the mutated position. Further details related to this* in silico* mutagenesis procedure, including descriptive figures, may be accessed from our previously published work [8, 14].

## 3. Software Implementation

Included in the main AUTO-MUTE 2.0 folder are five programs, analogous to their respective counterparts on the online server and executable from the command-line:

- stability_changes_ddG.pl,
- stability_changes_ddG_H2O.pl,
- stability_changes_dTm.pl,
- activity_changes.pl,
- human_nsSNPs.pl.

Entering the command “perl <program_name>” returns detailed information, including proper usage of required and optional flags (-t, -f, -m, and -o) after the program name. All PDB files of protein chains for which mutant predictions are requested should first be saved in the main folder and named pdbXXXX.ent, where XXXX is the 4-character PDB ID, a process that can be performed automatically by the program (-t 0) or manually by the user ahead of time (-t 1). The main folder should also contain a text file of the prediction requests (-f <mutant_data_filename>), such that the data items on each line describe a single mutant and are separated by spaces or tabs (e.g., “3phvA D25E 25 7” or “1gvpA S20T”; see README file in the main folder for additional details). Each of the three stability change predictors offers two supervised classification models (-m 0, -m 1) and two regression models (-m 2, -m 3) from which to select (see README file for details about the models); this flag is not applicable with the activity_changes.pl and human_nsSNPs.pl programs, each of which generates predictions with a single, distinct random forest (RF) classifier trained using published experimental data. Lastly, users can optionally specify an output file to be used for prediction results (-o <output_filename>); otherwise, results appear on the command-line screen (standard output). Table 1 provides supervised classification (RF) and tree regression (REPTree) predictions obtained for a sample text file of protein mutants using the program stability_changes_ddG.pl.

Three temporary files populate the main AUTO-MUTE 2.0 folder: dinputs.txt retrieves the protein chain C-alpha coordinates formatted as an input file for the Qhull software package, doutputs.txt stores the tessellation information that Qhull provides as output, and test_vector.arff contains the feature vectors for all the requested mutants in a file format that can be processed for prediction by the models we trained using the Weka software package. The AUTO-MUTE 2.0 programs should be executed consecutively, as parallel runs will yield errors stemming from simultaneous use of these temporary files by more than one program. Two subdirectories are found in the main folder: one for extensive support codes used in deriving the mutant feature vectors that appear in the test_vector.arff file and the other for housing the trained models that are used in generating mutant predictions from these vectors. Training sets, model performance data, and detailed prediction comparisons with related methods that demonstrate an improvement using our approach are published elsewhere [7, 8, 15, 16], although this material is also available as supporting documentation on the AUTO-MUTE website, accessible from the “details” link provided on the input page for each prediction tool.

## 4. New Features Integrated into AUTO-MUTE 2.0

As a response to the limited capability of the web server, and in support of the evolving “big data” environment, the number of predictions from a single run of any program in the new AUTO-MUTE 2.0 stand-alone software is now unrestricted and depends only on the quantity of mutants requested in the submitted text file. These single residue replacements all may correspond to a single protein chain, or they may involve any number of distinct structures, so long as each line in the text file submitted to the program provides the necessary information about the requested mutant. As fully described in the README file located in the main AUTO-MUTE 2.0 folder, all five programs require that each line includes the 4-character PDB ID with an attached letter indicating the protein chain (e.g., 3phvA), as well as the requested mutation (e.g., D25E) in the form (native residue)(position number)(replacement residue). An important exception applicable only to the two programs stability_changes_ddG.pl & stability_changes_ddG_H2O.pl requires that temperature (°C) and pH for each mutant also be included in the text file (e.g., 3phvA D25E 25 7), where 25°C and pH = 7 are appropriate default values (Table 1).

Next, single residue substitutions in personal (non-PDB) model structures can now be predicted and may be included in the same text file with mutations occurring in PDB structures; however, for proper processing by the programs, they should be (1) given randomly selected 4-character IDs and named pdbXXXX.ent similar to PDB files, (2) formatted identical to PDB files with respect to their HELIX, SHEET, and ATOM lines, and (3) placed in the main AUTO-MUTE 2.0 folder and used with the manual (-t 1) option when running the programs. Details are available from the PDB about how to appropriately format the HELIX and SHEET lines (http://www.wwpdb.org/documentation/format32/sect5.html) as well as the ATOM lines (http://www.wwpdb.org/documentation/format32/sect9.html#ATOM).

Third, any NMR structure file in the PDB that contains multiple models can now be submitted, and the C-alpha atomic coordinates provided in Model 1 are those used for tessellating the corresponding protein. This decision is based on the fact that first model in such PDB files is reserved for the one representative model that is closest to the average model (http://deposit.rcsb.org/depoinfo/print_nmr.html); hence, Model 1 provides the most reliable set of coordinates for generating the most accurate predictions using our methodology. With respect to “minimized average structure” NMR files in the PDB, these pose no difficulty for either the online server or AUTO-MUTE 2.0 since the files each contain a single set of atomic coordinates for their respective proteins. Lastly, protein chains with multiple conformation C-alpha atoms, as well as those with sequentially numbered residues in the ATOM lines that start with a negative number, are now no longer excluded and can be processed by AUTO-MUTE 2.0.

On the other hand, the following restrictions have remained in place and will generate error messages: protein chains cannot be used if their structural coordinate files contain nonsequential residue numbering (gaps) or nonstandard amino acids, and predictions for mutated positions connected to fewer than six neighbors cannot be processed. Any protein chain with a gap has a sequential run of missing residues in its 3D structure, making C-alpha coordinates unavailable for these positions; hence, the Delaunay tessellation of such a structure will be faulty, consisting of numerous tetrahedra that falsely identify residue quadruplets at their vertices as nearest neighbors and leading to erroneous predictions. Researchers encountering such PDB structures may overcome this restriction by modeling the gapped segments and using their modified structure files, which are sequentially numbered and contain C-alpha coordinates for all residues. Nonstandard amino acids cannot be incorporated due to the fact that our computational mutagenesis methodology relies on a four-body statistical potential defined over a standard 20-letter protein alphabet (i.e., there are no scores for residue quadruplets that contain nonstandard amino acids). And any mutated position with fewer than six neighbors is relatively well isolated in its local environment, violating a fundamental condition in our method for deriving a complete feature vector for the mutant.

## 5. System Requirements

Both Perl (including the CPAN library) and Java are freely available and should be installed prior to running AUTO-MUTE 2.0. Cygwin users should initially install the Qhull software from the online package list (http://cygwin.com/cygwin/packages/); Qhull already comes bundled with the AUTO-MUTE 2.0 downloads for PC and Mac. The AUTO-MUTE 2.0 folder contains the Weka file weka.jar, to which the computer CLASSPATH environment variable must point, as described on the Weka website (http://weka.wikispaces.com/CLASSPATH). Qhull and Weka are freely available software tools used without modification by AUTO-MUTE 2.0. Finally, web access is required for automatic (-t 0) PDB file downloads.

## 6. Conclusion

In summary, AUTO-MUTE 2.0 is a stand-alone software package for structure-based prediction of protein functional changes upon single residue replacements. A number of additional features have been integrated into this new portable alternative to our original web-based predictors, based on substantial feedback provided by members of the scientific community. One critical enhancement now allows for the batch prediction of an unrestricted number of mutants (associated with one or multiple proteins) from a single run of a program, hence addressing needs of researchers conducting protein mutagenesis studies in a “big data” environment. Additional modifications made to the original programs now also enable predictions to be obtained for mutants associated with a wider array of admissible protein structure files, including all personal (non-PDB) models; NMR-derived structures consisting of multiple models; structure files for which one or more amino acid residues have multiple conformation C-alpha atoms; and structure files with sequentially numbered residues that begin with a negative number. Finally, all of the original Java and PHP codes used for the online server were rewritten and modified for AUTO-MUTE 2.0 using Perl, a language especially popular among researchers in the biological sciences having only a modest programming background. The programs were written in a straightforward manner that excluded the object-oriented paradigm, thus offering bench scientists an opportunity to easily comprehend the underlying logic and to even reuse or modify portions of the Perl codes in accordance with their particular needs.

## Figures and Tables

**(a):** 

mutants.txt			
3phvA	D25E	25	7
3phvA	T31A	25	7
4lyzA	A31V	25	7
4lyzA	D101R	25	7
1benD	L15Q	25	7
1rn1C	Q25K	25	7
1g35B	H69N	25	7
1g35B	L90M	25	7
1pohA	K49E	25	7

**(b):** 

C:∖AutoMute2>perl stability_changes_ddG.pl -t 0 -f mutants.txt -m 0 -o sample_output_ddG_RF.txt											

sample_output_ddG_RF.txt											

PDB_ID	Chain	Mutation	Stability	Confid.	Temp.	pH	Vol.	sT	Loc.	Num.	SS

3phv	A	D25E	Decreased	0.62	25	7	14.6	0.16	B	0	C
3phv	A	T31A	Decreased	0.64	25	7	14.1	0.14	B	0	S
4lyz	A	A31V	Increased	0.86	25	7	19.5	0.10	B	0	H
4lyz	A	D101R	Increased	0.85	25	7	17.7	0.18	S	6	C
1ben	D	L15Q	Decreased	0.90	25	7	10.9	0.13	U	1	H
1rn1	C	Q25K	Increased	0.96	25	7	8.7	0.14	S	6	H
1g35	B	H69N	Decreased	0.67	25	7	15.2	0.18	S	4	S
1g35	B	L90M	Decreased	0.85	25	7	18.9	0.13	B	0	H
1poh	A	K49E	Increased	1.00	25	7	10.1	0.12	S	4	H

**(c):** 

C:∖AutoMute2>perl stability_changes_ddG.pl -t 1 -f mutants.txt -m 2 -o sample_output_ddG_REPTree.txt										

sample_output_ddG_REPTree.txt										

PDB_ID	Chain	Mutation	ddG	Temp.	pH	Vol.	sT	Loc.	Num.	SS

3phv	A	D25E	−1.95	25	7	14.6	0.16	B	0	C
3phv	A	T31A	−1.38	25	7	14.1	0.14	B	0	S
4lyz	A	A31V	1.23	25	7	19.5	0.10	B	0	H
4lyz	A	D101R	0.24	25	7	17.7	0.18	S	6	C
1ben	D	L15Q	−1.64	25	7	10.9	0.13	U	1	H
1rn1	C	Q25K	0.61	25	7	8.7	0.14	S	6	H
1g35	B	H69N	−0.60	25	7	15.2	0.18	S	4	S
1g35	B	L90M	−0.90	25	7	18.9	0.13	B	0	H
1poh	A	K49E	1.37	25	7	10.1	0.12	S	4	H
